# Extreme Concentrations of Nitric Oxide Control Daytime Oxidation and Quench Nocturnal Oxidation Chemistry in Delhi during Highly Polluted Episodes

**DOI:** 10.1021/acs.estlett.3c00171

**Published:** 2023-05-03

**Authors:** Beth S. Nelson, Daniel J. Bryant, Mohammed S. Alam, Roberto Sommariva, William J. Bloss, Mike J. Newland, Will S. Drysdale, Adam R. Vaughan, W. Joe F. Acton, C. Nicholas Hewitt, Leigh R. Crilley, Stefan J. Swift, Pete M. Edwards, Alastair C. Lewis, Ben Langford, Eiko Nemitz, Shivani, Ranu Gadi, Bhola R. Gurjar, Dwayne E. Heard, Lisa K. Whalley, Ülkü A. Şahin, David C. S. Beddows, James R. Hopkins, James D. Lee, Andrew R. Rickard, Jacqueline F. Hamilton

**Affiliations:** † Wolfson Atmospheric Chemistry Laboratories, Department of Chemistry, University of York, Heslington, York YO10 5DD, U.K.; ‡ School of Biosciences, University of Nottingham, Sutton Bonington, Leicestershire LE12 5RD, U.K.; § School of Geography, Earth and Environmental Sciences, University of Birmingham, Birmingham B15 2TT, U.K.; ∥ National Centre for Atmospheric Science, University of York, Heslington, York YO10 5DD, U.K.; ⊥ Lancaster Environment Centre, Lancaster University, Lancaster LA1 4YW, U.K.; # UK Centre for Ecology and Hydrology, Penicuik, Midlothian, Edinburgh EH26 0QB, U.K.; @ Department of Applied Sciences and Humanities, Indira Gandhi Delhi Technical University for Women, Delhi 110006, India; ∇ Indian Institute of Technology, Roorkee, Uttarakhand 247667, India; ■ School of Chemistry, University of Leeds, Leeds LS2 9JT, U.K.; □ National Centre for Atmospheric Science, University of Leeds, Leeds LS2 9JT, U.K.; ● Istanbul University-Cerrahpasa, Engineering Faculty, Environmental Engineering Department, Avcilar, 34320 Istanbul, Turkey; ○ National Centre for Atmospheric Science, University of Birmingham, Birmingham B15 2TT, U.K.

**Keywords:** air quality, atmospheric oxidation chemistry, nocturnal atmospheric chemistry, ozone, VOC

## Abstract

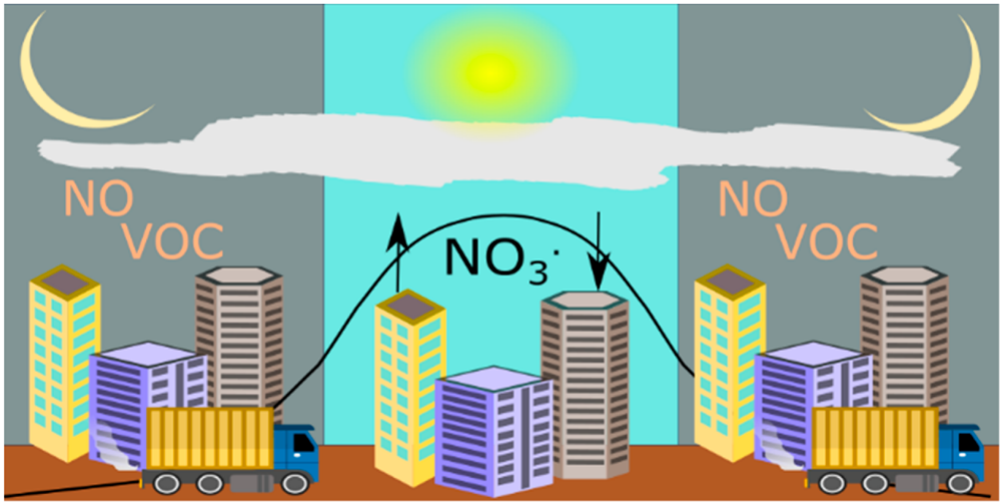

Delhi, India, suffers from periods of very poor air quality, but little is known about the chemical production of secondary pollutants in this highly polluted environment. During the postmonsoon period in 2018, extremely high nighttime concentrations of NO_x_ (NO and NO_2_) and volatile organic compounds (VOCs) were observed, with median NO_x_ mixing ratios of ∼200 ppbV (maximum of ∼700 ppbV). A detailed chemical box model constrained to a comprehensive suite of speciated VOC and NO_x_ measurements revealed very low nighttime concentrations of oxidants, NO_3_, O_3_, and OH, driven by high nighttime NO concentrations. This results in an atypical NO_3_ diel profile, not previously reported in other highly polluted urban environments, significantly perturbing nighttime radical oxidation chemistry. Low concentrations of oxidants and high nocturnal primary emissions coupled with a shallow boundary layer led to enhanced early morning photo-oxidation chemistry. This results in a temporal shift in peak O_3_ concentrations when compared to the premonsoon period (12:00 and 15:00 local time, respectively). This shift will likely have important implications on local air quality, and effective urban air quality management should consider the impacts of nighttime emission sources during the postmonsoon period.

## Introduction

Delhi, India, is one of the most polluted megacities in the world and has previously been shown to have levels of numerous air pollutants such as particulate matter (PM), NO_x_ (NO and NO_2_), O_3_, SO_2_, and CO significantly above World Health Organization (WHO) guidelines.^1−4^ High concentrations have led to an increase in morbidity and premature mortality, leading to economic losses.^5−7^ Exposure to air pollution has been shown to reduce life expectancy on average by six years in Delhi.^8^ Previous studies have highlighted significant amounts of premature mortality within Delhi, with estimates of up to 12 000 deaths per year associated with poor air quality.^3^

Measured concentrations of volatile organic compounds (VOCs) and NO_x_ in Delhi are among the highest recorded in a polluted urban environment, on a scale similar to those observed in Los Angeles in the 1970s.^9^ The concentrations of pollutants are especially high during the winter periods, largely due to emissions from the burning of crop residues, domestic heating, cooking, along with local meteorology.^10−17^ High NO_x_ concentrations have been observed throughout the year,^2^ with the highest observed during winter, and nighttime concentrations on average of >100 ppbV.^18,19^ Within Delhi, the transport sector is the dominant source of NO_x_ emissions, accounting for 66–74%.^20^ Of these transport emissions, heavy goods vehicles (HGVs) make the largest contribution (>50%).^21^ To combat transport pollution, the Government of National Capital Territory of Delhi introduced prohibitive regulations impacting the transport of goods into and out of the city. Prohibitions on the movement of goods vehicles vary across Delhi but generally restrict HGVs during peak times for passenger vehicles [07:00–11:00 and 17:00–23:00 Indian Standard Time (IST)].^22^ These daytime restrictions lead to increased HGV movement at night, and thus increased nighttime emissions. Delhi also suffers from unfavorable meteorological conditions, with shallow boundary layer heights and low wind speeds leading to stagnant conditions, especially during the winter. This, in turn, allows for the accumulation of pollutants at night, exacerbated by the high emission rates.^23^

The nature of radical chemistry at night, when pollution is high, may have significant consequences on the subsequent daytime oxidation chemistry and secondary pollution formation. A recent study by Bryant et al. presented evidence of the removal of nighttime NO_3_ radicals by NO in Delhi via a decrease in the concentration of isoprene-derived nitrooxy-organosulfate (NOSi) species produced via NO_3_ oxidation chemistry.^24^ In addition, a study by Nelson et al., corroborated by Chen et al., found central Delhi to be VOC-limited with respect to O_3_ production and significant titration of O_3_, owing to very high NO concentrations at night during the winter periods.^18,19^ The ratio of NO_x_ to non-methane VOCs has also been shown to impact rates of secondary organic aerosol formation.^25^ These studies have highlighted the importance of reducing levels of VOCs alongside those of NO_x_, to mitigate both O_3_ and PM formation. Our findings build on the work by Nelson et al. and Bryant et al. by focusing on the role NO plays in regulating OH and NO_3_ radical concentrations within Delhi.^19,24^ The unique chemistry investigated in this study deviates from our typical understanding of nocturnal radical oxidation chemistry and has important policy implications, highlighting the need to reduce nighttime NO_x_ emissions alongside VOC emissions, to not further exacerbate PM and O_3_ concentrations through secondary formation.

## Methodology

### Description of the Site

The APHH-India Delhi Flux campaigns took place during the premonsoon (May 28–June 5, 2018) and postmonsoon (October 4–November 5, 2018) periods. The primary site was located on the campus of the Indira Gandhi Delhi Technical University for Women in Delhi. The campus is situated close to major roads and highways, including National Highway 44 (0.3 km east). The campus has some green areas and is mostly pedestrianized with low-level traffic activity from delivery cars, taxis, and auto rickshaws. Observations were made from the roof of a one-story building (∼5 m) and included levels of VOCs, o-VOCs, NO_x_, CO, SO_2_, O_3_, and HONO, photolysis rates, PM, and meteorological measurements. Further details on the site can be found in ref (19).

### VOC Measurements

VOCs were measured via a dual-channel gas chromatography flame ionization detector (DC-GC FID, C_2_–C_8_), a two-dimensional GC flame ionization detector (GC × GC-FID, C_6_–C_13_), and a proton transfer reaction time-of-flight mass spectrometer with a quadrupole ion guide (PTR-QiTOF, Ionicon Analytik, Innsbruck, Austria). The two GC instruments shared an inlet, located 5 m above ground level. The sample line from the inlet to the laboratory was made from ^1^/_2_ in. (outside diameter) perfluoroalkoxy (PFA) and was heated. VOCs were calibrated using standard cylinders containing a variety of VOCs. Further details can be found in refs (19) and (26).

### Measurements of NO_x_, O_3_, and HONO

Measurements of nitrogen oxides (NO and NO_2_) were made using a dual-channel chemiluminescence instrument (Air Quality Designs Inc.) that was calibrated every 2–3 days throughout the campaign using standard gas cylinders from the National Physical Laboratories in the U.K. O_3_ was measured using an O_3_ analyzer (49i, Thermo Scientific). The instrument setup and calibration methodology are as described by Squires et al.^27^ HONO was measured using a long-path absorption photometer (LOPAP) with baseline measurements taken at regular intervals (8 h). Further details along with measurement descriptions for the aerosol surface area, photolysis rates, and meteorological data can be found in refs (19) and (22).

### Description of the Model

Two campaign-tailored zero-dimensional chemical box models (premonsoon and postmonsoon) incorporating a subset of the Master Chemical Mechanism (MCM version 3.3.1)^28,29^ were used to investigate radical production chemistry, utilizing the AtChem2 modeling toolkit.^30^ The pre- and postmonsoon models were constrained to the measured concentrations of ambient VOCs (57 and 86, respectively), NO_x_, CO, O_3_, SO_2_, aerosol surface area (postmonsoon only), 34 photolysis rates derived from the measured photolysis frequencies of *j*(O^1^D), *j*(NO_2_), and *j*(HONO), temperature, pressure, and relative humidity. The premonsoon model was constrained to a reduced range of VOCs due to the concentration of some species being below the instrumental limits of detection. Measurement data were averaged or linearly interpolated to 15 min time resolution prior to inclusion in the models. The first 24 h of measurements were repeated for 3 days prior to the full campaign time series. These modeled data were then discarded before analysis to ensure radical spin-up was achieved. Measured species not described in the MCM were incorporated using a surrogate mechanism (Table S1), chosen on the basis of the structural similarity to the species of interest (more details in ref (19)). For these species, reaction rates with OH, HO_2_, and NO_3_ were set to values found in the IUPAC evaluated chemical kinetics database.^31,32^ For both campaigns, a fixed deposition rate of 1.2 × 10^–5^ s^–1^ was applied to all model-generated species, resulting in a lifetime with respect to dry deposition of ∼24 h. Where vertical velocity data were available (postmonsoon only), the concentration of HONO was adjusted to account for variations within the vertical profile due to its very short lifetime relative to that of VOCs, and the atmospheric mixing time.^33^ This adjustment is particularly important for the postmonsoon period, given the very high levels of HONO measured at ground level. The model also accounted for aerosol uptake of HO_2_. A more detailed description of the adjustments to HONO observations, calculation of photolysis rates, and HO_2_ uptake is presented in ref (19).

## Results

**Figure 1:**
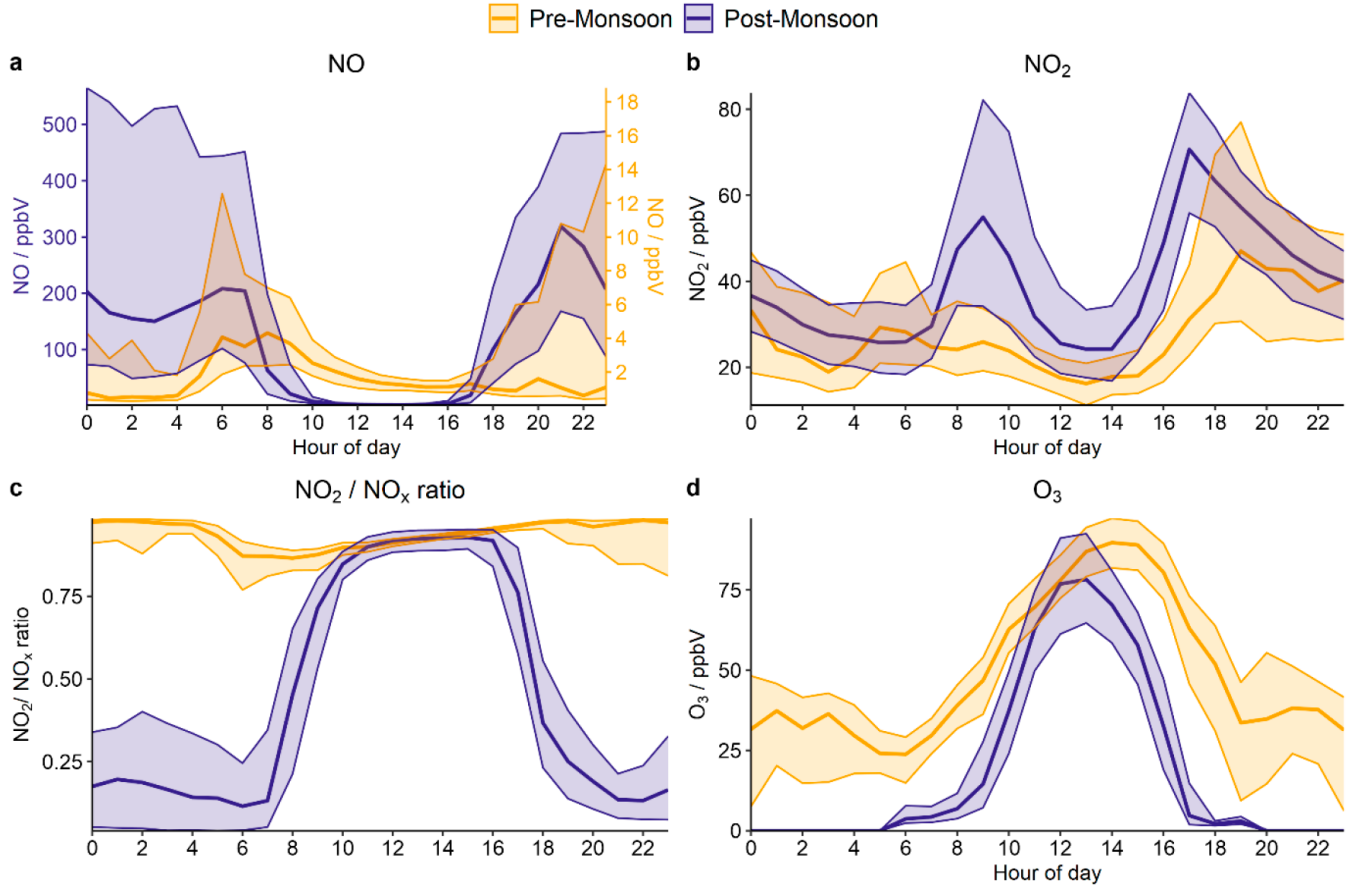
Median diel profiles of (a) NO, (b) NO_2_, (c) NO_2_:NO_x_ ratio, and (d) O_3_ measured during the premonsoon (yellow) and postmonsoon (purple) campaigns. Note that pre/postmonsoon NO values are plotted on separate *y*-axes owing to the large differences in observed mixing ratios. The shaded region represents the interquartile range.

The median diel profiles of NO mixing ratios observed during both campaigns are shown in Figure 1. The observed NO levels are at a minimum during the day, likely driven by differences in emissions but also corresponding to the period during which the planetary boundary layer height (PBLH) is greatest (Figure S1). The reaction of NO with O_3_ converts NO to NO_2_, resulting in NO mixing ratios in the afternoon of ∼1–2 ppbV. The NO_2_:NO_x_ ratio in the afternoon is ∼0.92–0.96, indicating a high NO_2_ conversion rate under strong photochemically active conditions. In the evening, a shallow nocturnal boundary layer is formed (Figure S1), leading to a rapid increase in the mixing ratio of NO. The nighttime PBLH minimum is much lower during the postmonsoon period (median PBLH is 33 m, vs 210 m during the premonsoon), leading to a median nocturnal mixing ratio of 200 ppbV for NO, ∼400 times higher than during the premonsoon period (∼0.50 ppbV). On some nights, extremely high NO mixing ratios were observed, up to a maximum of ∼700 ppbV.^19^ Simultaneously, O_3_ mixing ratios decreased due to reaction with NO, with median nocturnal O_3_ mixing ratios in the postmonsoon period of <2 ppbV, compared to ∼30 ppbV during the premonsoon period (Figure 1). However, measured nighttime O_3_ concentrations should be treated with caution as chemical interference from, for example, aromatic compounds can cause small positive artifacts.^34^ The extremely high levels of NO at night during the postmonsoon period are likely to entirely remove all nighttime O_3_ through chemical titration, evidenced by model simulations of O_3_ (Figure S2). The lifetime of NO in a typical urban atmosphere is relatively short, such that NO_2_:NO_x_ ratios can increase significantly within tens of seconds after emission.^35^ Overnight, the postmonsoon NO_2_:NO_x_ ratio was ∼0.15 on average, indicative of direct combustion emissions of NO.^36^ The observed ratio is comparable to previous observations in road tunnels, indicating a lack of NO oxidation at night in Delhi once all of the O_3_ produced during the day has been consumed.^37^

**Figure 2:**
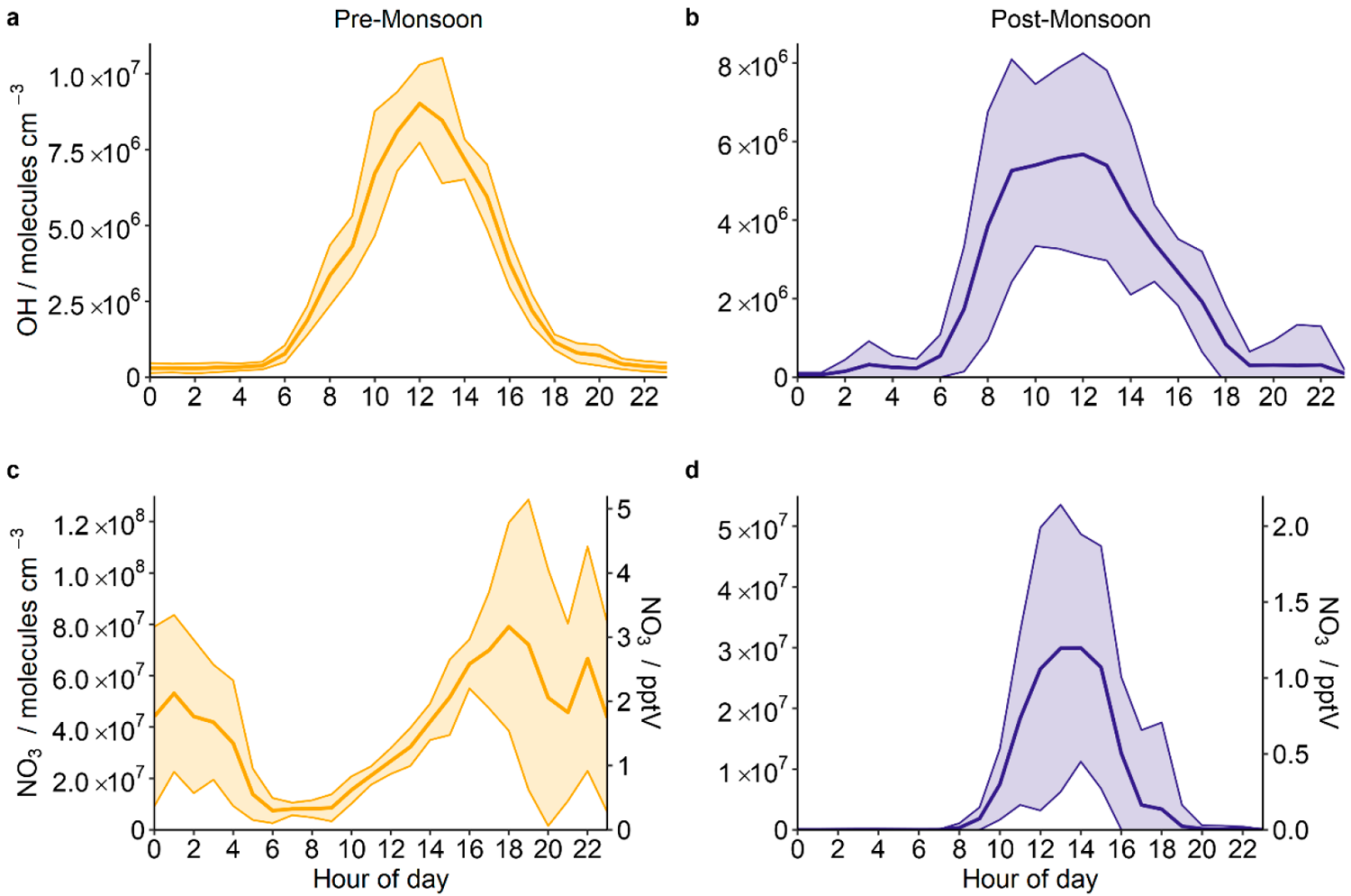
Mean diel variations of modeled OH and NO_3_ concentrations across the (a and c) premonsoon and (b and d) postmonsoon periods. The shaded band represents one standard deviation from the mean. Note that the *y*-axis scales on all plots are distinct.

NO_x_ plays a key role in the production of all three major oxidants (OH, NO_3_, and O_3_) in polluted atmospheres and therefore impacts radical production and loss.^32,38^ VOC oxidation is predominantly driven by OH and O_3_ during the day and NO_3_ and O_3_ at night. We used an observationally constrained zero-dimensional chemical box model to predict short-lived radical oxidant concentrations during the pre- and postmonsoon periods. During the premonsoon period, the OH radical followed the expected diel profile, peaking at solar noon with a concentration of ∼9 × 10^6^ molecules cm^–3^ (Figure 2a). During the postmonsoon period, the OH radical concentrations peak in the morning, reaching a maximum of 6 × 10^6^ molecules cm^–3^ at 9:00 IST which begins to decrease only after 13:00 IST (Figure 2b). NO_3_ showed more significant seasonal differences. During the premonsoon period, the modeled NO_3_ concentrations peaked just after sunset (17:00–19:00 IST, 8 × 10^7^ molecules cm^–3^, 3.2 pptV) and remained high overnight before rapidly decreasing after sunrise due to photolysis (Figure 2c). NO_3_ concentrations then increased throughout the day due to NO concentrations being suppressed through reactions with O_3_, reducing this important NO_3_ sink. During the postmonsoon period, modeled NO_3_ concentrations peaked between 12:00 and 14:00 IST, at a concentration comparable to that observed at the same time during the premonsoon period (∼3 × 10^7^ molecules cm^–3^, 1.2 pptV) (Figure 2d). A comparison between NO_3_ photolysis rates and modeled NO_3_ suggests that daytime peaks in NO_3_ are not significantly influenced by haze events (see Figure S3). The differences seen in the NO_3_ diel profiles are striking, with the extreme levels of NO at night rapidly scavenging any NO_3_ that is formed during the postmonsoon period. It should be noted that the model does not account for N_2_O_5_ uptake, though we expect the impact of this to be minimal as our modeled N_2_O_5_ midday peak is small (<10 pptV), relative to the daytime concentration of NO (>1.8 ppbV). The impact of N_2_O_5_ uptake on modeled NO_3_ concentrations is investigated in more detail in ref (39).

**Figure 3:**
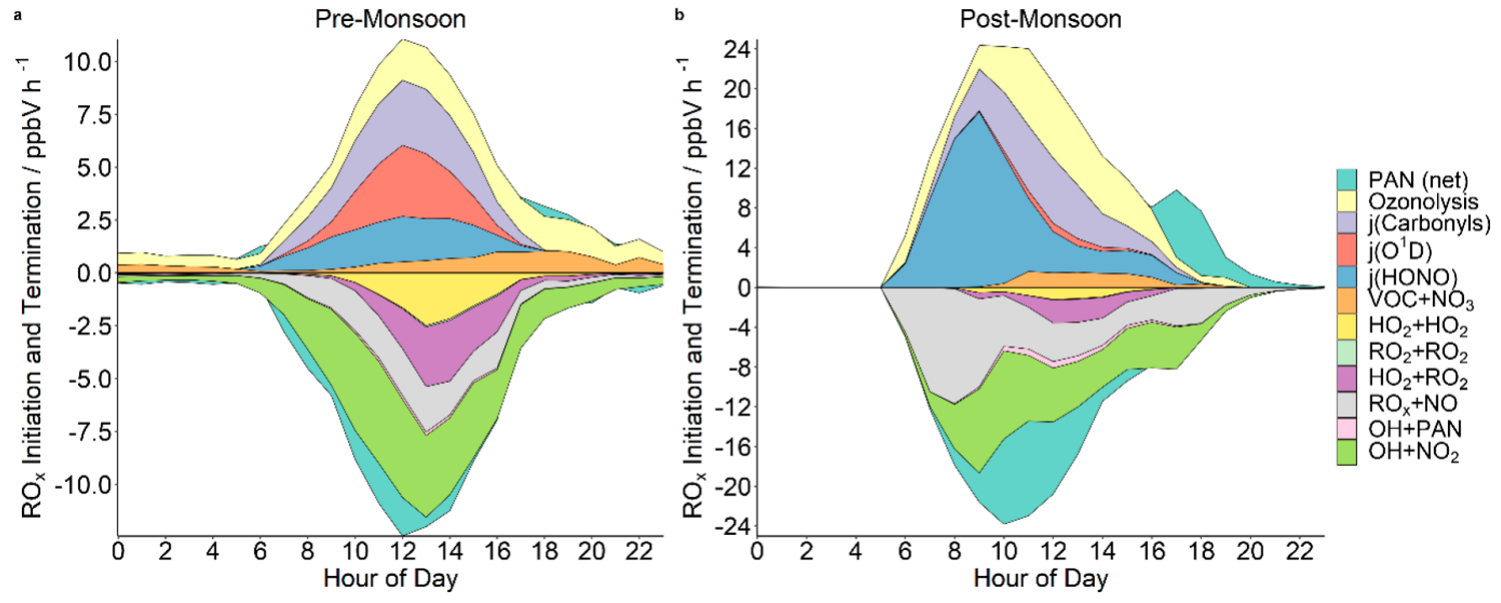
Stacked area plots showing the average modeled diel production (initiation, positive) and loss (termination, negative) rates for OH + HO_2_ + RO_2_ (RO_x_) radicals during the (a) premonsoon and (b) postmonsoon campaigns, under zero oxidant nighttime conditions during the postmonsoon period. PAN (net) refers to the PAN ⇌ CH_3_CO_3_ + NO_2_ equilibrium, where CH_3_CO_3_ is being formed in the positive direction (PAN loss) and being lost in the negative direction (PAN formation). Note the magnitudinal difference between the *y*-axes scales on the two plots.

To further investigate the drivers of Delhi’s radical chemistry, the rates of radical production and loss were determined for each campaign. The average modeled diel rates of production (initiation, positive values) and loss (termination, negative values) of OH, HO_2_, and RO_2_ (RO_x_) radicals are shown as a stacked plot in Figure 3 for both campaigns. The pre- and postmonsoon percentage contribution to radical initiation/termination (by time of day) for each source is shown in Figures S4 and S5. During the premonsoon period, the radical production rate is symmetrical around noon (maximum of ∼10 ppbV h^–1^), with roughly similar contributions from ozonolysis, photolysis of carbonyls, photolysis of O_3_ (*j*(O^1^D)), and HONO photolysis. VOC + NO_3_ reactions start to make a significant contribution to radical initiation in the late afternoon (Figure 3). In contrast, there is a large burst of RO_x_ radical formation after sunrise during the postmonsoon period (maximum of ∼24 ppbV h^–1^), more than double the radical production observed during the premonsoon period (Figures S6). This is largely driven by the photolysis of high levels of HONO (∼12 ppbV) remaining from the previous night (Figure S7). The morning radical production peak is sustained into the afternoon by ozonolysis and carbonyl photolysis, whereas the photolysis of O_3_ plays an only minor role (Figures S4 and S5). The postmonsoon morning burst in radical production results in a temporal shift in the peak O_3_ concentration, from 15:00 IST during the premonsoon period to 13:00 IST during the postmonsoon period, although overall the maximum O_3_ mixing ratios are similar between the two campaigns. The temporal O_3_ peak shift observed in the measurements was also seen by the model in a simulation in which O_3_ was unconstrained, with modeled O_3_ also peaking around midday.

A similar pattern is seen for radical loss processes, with the premonsoon period showing a symmetrical diel profile, with OH + NO_2_, RO_2_ + HO_2_, RO_x_ + NO, and HO_2_ + HO_2_ all acting as important termination reactions. The postmonsoon radical losses are driven by high NO levels present after sunrise with the RO_x_ + NO reaction dominating the termination reactions initially. The RO_x_ + NO term is a combination of OH + NO (=HONO) and RO_2_ + NO (=RONO_2_) terminating loss routes and is dominated by OH + NO loss during the pre- and postmonsoon periods (∼60% and ∼86%, respectively). During the premonsoon period, RO_2_ products derived from isoprene and monoterpenes make the next largest contributions (∼20%). During the postmonsoon period, dodecane-derived RO_2_ makes the next largest contribution (∼2%). All other pathways in both campaigns make minor contributions to the overall RO_x_ + NO termination term, with OH + NO_2_ and net peroxyacetyl nitrate (PAN) formation becoming increasingly important by 9:00 IST. This balance between radical production and loss, driven by the high levels of NO_x_, likely stops O_3_ concentrations during the postmonsoon period reaching the increased levels observed in Los Angeles in the 1970s.

## Discussion

We conclude that the very high NO levels observed in Delhi at night during the postmonsoon period quench nocturnal oxidation chemistry, evidenced by very low levels of measured O_3_ and modeled NO_3_ present and extremely low model radical initiation rates. This also suggests a concurrent lack of N_2_O_5_ formation at night. Therefore, even though very high levels of chloride (Cl^–^) in the particle phase have been reported in Delhi,^40^ limited nighttime ClNO_2_ formation, which is an important photochemical Cl radical reservoir, is likely to occur. The lack of nocturnal oxidation chemistry can also be confirmed using the VOC observations. Very high nighttime levels of monoterpenes were observed (mean of 2.5 ppbV), with eight nights having a peak over 5 ppbV (Figure S8).^19^ Species such as α-phellandrene, α-terpinene, and terpinolene are extremely reactive toward OH, O_3_, and NO_3_ (see Table S2) but did not accumulate overnight, reaching maximum mixing ratios of 120–350 pptV. During the premonsoon period, nighttime concentrations of these reactive monoterpenes were usually below the instrument detection limits and so were not included in the premonsoon model. The absence of nighttime oxidation chemistry is further corroborated by a study by Cash et al., which reported a reduction in oxidized aerosol levels in Delhi during the postmonsoon period at night, alongside increased levels of hydrocarbon-like organic aerosol (HOA).^41^ Together, these model and measurement observations provide strong evidence for a deviation from the typical nocturnal oxidation chemistry observed in other highly polluted urban atmospheres. More details about the observed VOC concentrations and diel profiles can be found in refs (19) and (42).

The observed postmonsoon overnight accumulation of a highly reactive mix of NO_x_ and oxidized nitrogen species (NO_y_) and VOCs, in a very shallow boundary layer, has important consequences for the subsequent day’s photochemistry. These trapped emissions act as a reservoir of photochemical fuel leading to high radical formation rates, initially driven by nitrous acid photolysis in the morning, and efficient photochemical O_3_ formation, shifting the postmonsoon maximum O_3_ peak earlier (by 3 h compared to the premonsoon peak). The observed temporal shift in maximum O_3_ coinciding with solar noon supports the presence of highly active oxidation chemistry in the morning. As the sun rises and the nocturnal boundary layer collapses in the morning, some of the accumulated pollution will be ventilated away from the surface. However, we observed that the modeled radical initiation pathways during the morning were very efficient after these stagnation events.

Recent pollution abatement policies that restrict the movement of HGVs during the day may be exacerbating nighttime pollution leading to the modeled early morning burst in secondary pollutant formation initiated by photolysis at sunrise. We propose that pollution reduction policies in central Delhi should focus on diel emission reductions, as policies that move emissions from daytime to nighttime may not be effective at reducing O_3_ and may lead only to a temporal shift in its peak. Further work is needed to determine the impact of reducing nighttime emissions on changes to daytime exposure to secondary pollutants, and future policies should consider the emission profile and atmospheric chemistry across the entire 24 h period.

## Supporting Information Available

List of measured VOCs used in the model (Table S1); diel profiles for boundary layer height, HONO, and total monoterpenes (Figures S1, S7, and S8, respectively); O_3_ postmonsoon modeled versus measured normalized diel profiles (Figure S2); photolysis rate variations (Figure S3); atmospheric lifetimes of selected species (Table S2); hourly modeled contributions to radical initiation and termination during the postmonsoon period (Figures S4 and S5); and full model radical flux analysis (Figure S6) (PDF)
