# Supplementary material for: Extreme Concentrations of Nitric Oxide Control Daytime Oxidation and Quench Nocturnal Oxidation Chemistry in Delhi during Highly Polluted Episodes
Source: Environ Sci Technol Lett. 2023 May 3;10(6):520–7. doi: 10.1021/acs.estlett.3c00171 (PMC10275353; doi:10.1021/acs.estlett.3c00171)
Supplement: Supplementary file 1 — ez3c00171_si_001.pdf [file ez3c00171_si_001.pdf]

Supplement: Extreme concentrations of nitric oxide control daytime oxidation and quench nocturnal oxidation chemistry in Delhi during highly polluted episodes

Beth S. Nelson[1*], Daniel J. Bryant[1*], Mohammed S. Alam[2], Roberto Sommariva[3], William J. Bloss[3], Mike J. Newland[1], James R. Hopkins[1,4], Will S. Drysdale[1,4], Adam R. Vaughan[1], W. Joe F. Acton[5a], Leigh R. Crilley[3b], Stefan J. Swift[1c], Pete M. Edwards[1], Alastair C. Lewis[1,4], Ben Langford[6], Eiko Nemitz[6], Shivani[7], Ranu Gadi[7], Bhola R. Gurjar[8], Dwayne E. Heard[9], Lisa K. Whalley[9,10], Ülkü A. Şahin[11], David C. S. Beddows[3,12], James D. Lee[1,4], Andrew R. Rickard[1,4], Jacqueline F. Hamilton[1,4]

1. Wolfson Atmospheric Chemistry Laboratories, Department of Chemistry, University of York, Heslington, York, YO10 5DD, UK
2. School of Biosciences, University of Nottingham, Sutton Bonington, Leicestershire, LE12 5RD, UK
3. School of Geography, Earth and Environmental Sciences, University of Birmingham, Birmingham, B15 2TT, UK
4. National Centre for Atmospheric Science, University of York, Heslington, York, YO10 5DD, UK
5. Lancaster Environment Centre, Lancaster University, Lancaster, LA1 4YW, UK
6. UK Centre for Ecology and Hydrology, Penicuik, Midlothian, Edinburgh, EH26 0QB, UK
7. Department of Applied Sciences and Humanities, Indira Gandhi Delhi Technical University for Women, Delhi, 110006, India
8. Indian Institute of Technology, Roorkee, Uttarakhand, 247667, India
9. School of Chemistry, University of Leeds, Leeds, LS2 9JT, UK
10. National Centre for Atmospheric Science, University of Leeds, Leeds, LS2 9JT, UK
11. Istanbul University-Cerrahpasa, Engineering Faculty, Environmental Engineering Department, Avcilar, Istanbul, Turkey
12. National Centre for Atmospheric Science, University of Birmingham, Birmingham, B15 2TT, UK
[a]now at: School of Geography, Earth and Environmental Sciences, University of Birmingham, Birmingham, B15 2TT, UK
[b]now at: WSP Australia, Brisbane, Australia
[c] now at: J. Heyrovsky Institute of Physical Chemistry, Department of Chemistry of Ions in Gaseous Phase, Prague, Czech Republic
Correspondance email: *beth.nelson@york.ac.uk, *daniel.bryant@york.ac.uk

Table S1: List of surrogate VOCs included in model. Rate constants for additional species to those in the MCM can be found on the IUPAC Task Group on Atmospheric Chemical Kinetic Data Evaluation database (https://iupac.aeris-data.fr/, last accessed: December 2022), originally published in Atkinson et al., 2006, or in Atkinson and Arey, 2003.

Species	Surrogate species (Proxy Mechanism) used in the MCM
n-Tetradecane	*n*-Dodecane
n-Tridecane	*n*-Dodecane
1,2-Butadiene	1,3-Butadiene
Propyne	Acetylene
1,2,3,4-Tetramethylbenzene	1,2,3-Trimethylbenzene
1,2,3,5-Tetramethylbenzene	1,2,3-Trimethylbenzene
1,2,4,5-Tetramethylbenzene	1,2,4-Trimethylbenzene
1,2-Diethylbenzene	*o*-Ethyltoluene

1,2-Dimethyl-4-ethylbenzene	3,5-Dimethyl-1-ethylbenzene
1,3-Diethyl-2-ethylbenzene	3,5-Dimethyl-1-ethylbenzene
1,3-Diethylbenzene	*m*-Ethyltoluene
1,4-Diethylbenzene	*p*-Ethyltoluene
1-Methyl-3-propylbenzene	Propylbenzene
1-Methyl-4-propylbenzene	Propylbenzene
1-Methylpropylbenzene	Propylbenzene
2,3-Dimethyl-1-ethylbenzene	3,5-Dimethyl-1-ethylbenzene
2,4-Dimethyl-1-ethylbenzene	3,5-Dimethyl-1-ethylbenzene
2-Methylpropylbenzene	Propylbenzene
n-Butylbenzene	Propylbenzene
α-Phellandrene	α-Pinene
3-Carene	α-Pinene
Camphene	β-Pinene
m-Cymene	*m*-Ethyltoluene
Myrcene	Limonene
o-Cymene	*o*-Ethyltoluene
p-Cymene	*p*-Ethyltoluene
Sabinene	β-Pinene
Terpinolene	β-Pinene
α-Terpinene	α-Pinene
β-Ocimene	Limonene
γ-terpinene	α-Pinene

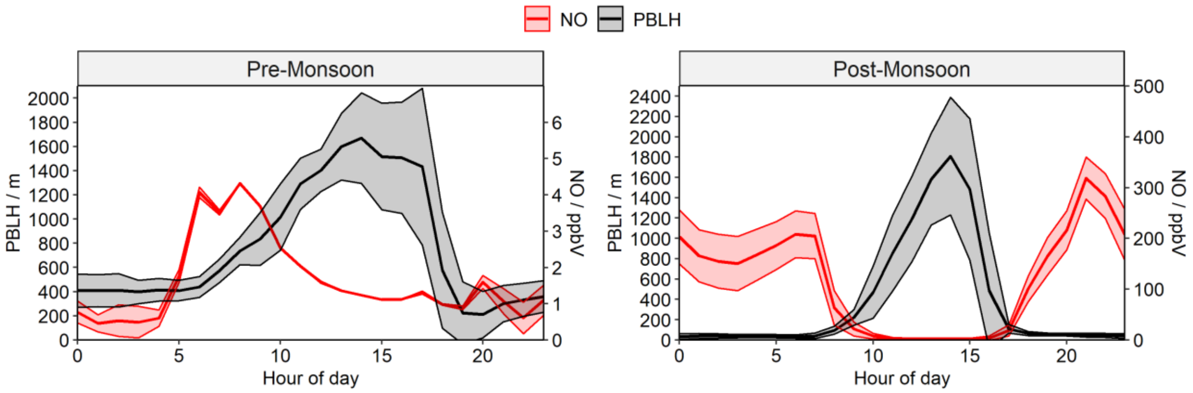

Figure S1: Mean diel planetary boundary layer height (PBLH, black) and NO mixing ratios (red). The coloured ribbon represents one standard deviation from the mean.

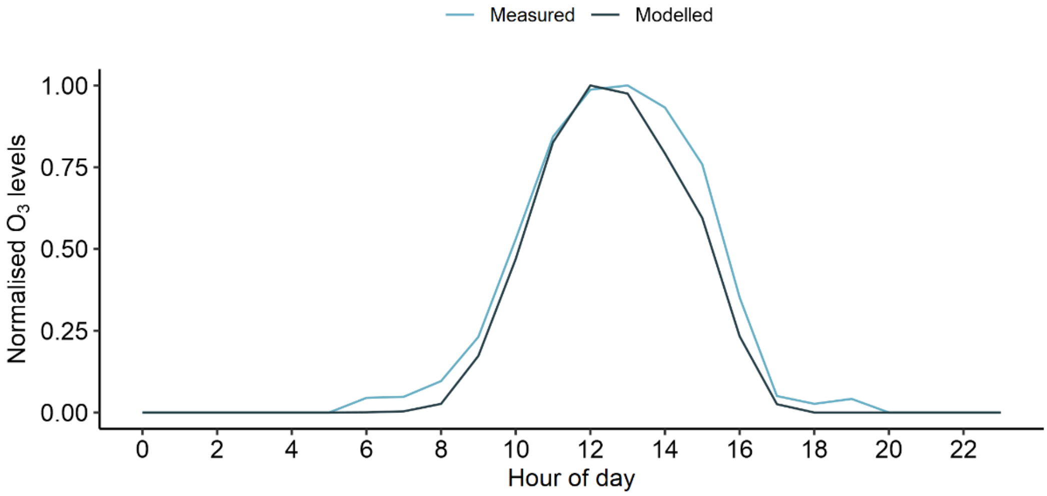

Figure S2: Normalised diel O_3 profiles observed in the Delhi post-monsoon period (light blue) and produced by the constrained chemical box model (dark blue).

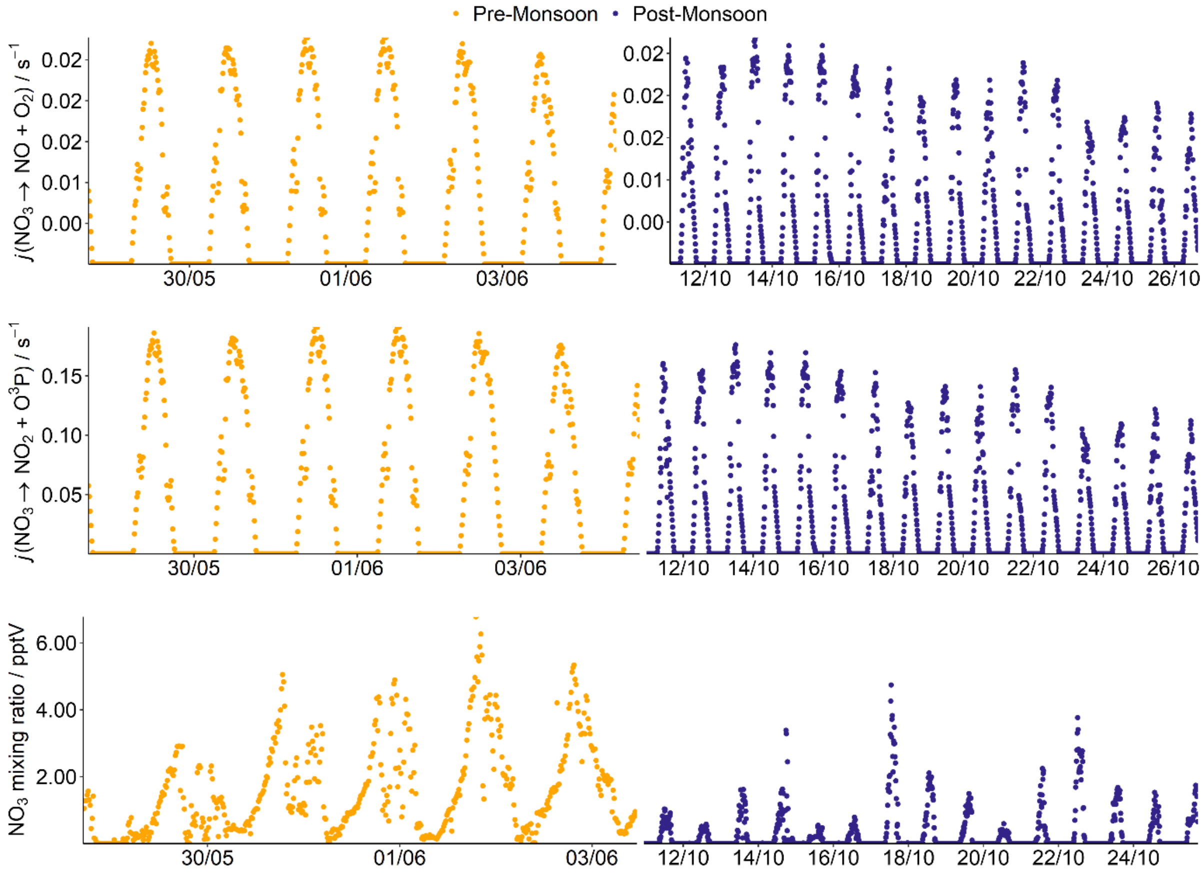

Figure S3: Diel variations in NO_3 photolysis rates compared to modelled NO_3 during the pre-monsoon (left) and post-monsoon (right) periods.

Table S2: Atmospheric lifetimes at 298 K with respect to reaction with OH, NO_3 and O_3 for a selection of VOCs. Assumed concentrations of OH, NO_3, and O_3 are 1.6 x 10^6, 3.5 x 10^8 and 7.5 x 10^{11} molecule cm^{-3} respectively, typical in urban environments. Preferred rate constants as reported by IUPAC were used (Atkinson et al., 2004).

VOC	Post monsoon mean / ppbV	Post monsoon max / ppbV	Lifetime via reaction with OH (298 K) / min	Lifetime for reaction via O_3 (298 K) / min	Lifetime via reaction with NO_3 (298 K) / s
a-phellandrene	0.07	0.35	33	23	39
a-terpinene	0.02	0.12	30	3.5	16
terpinolene	0.04	0.29	47	42	29

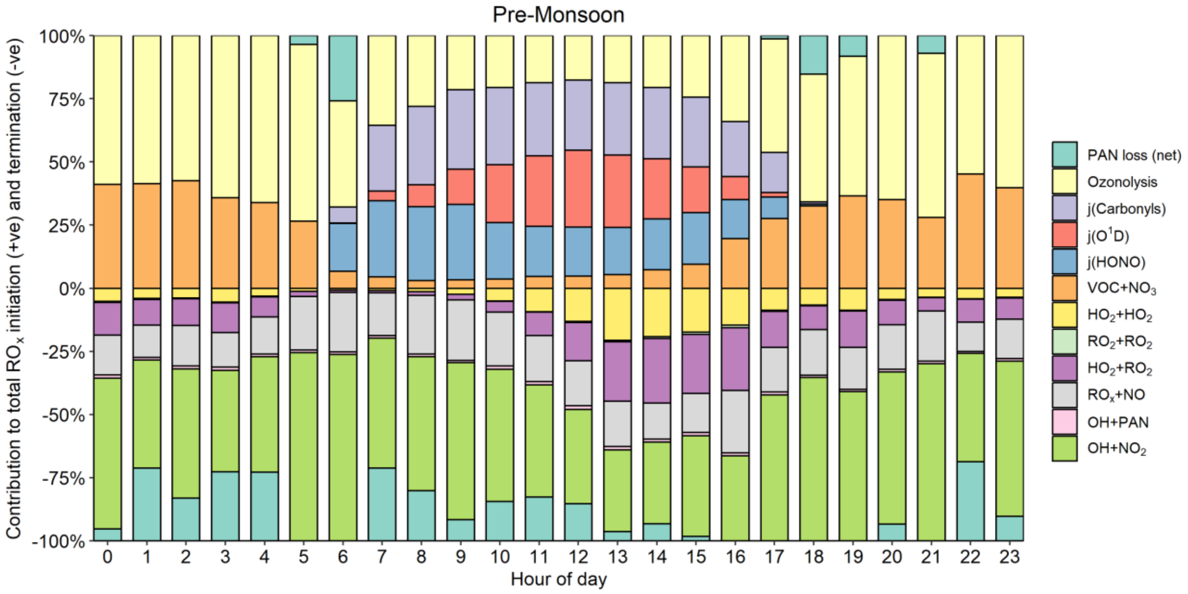

Figure S4: Bar chart showing the hourly averaged modelled contributions to total radical initiation (positive) and termination (negative) during the pre-monsoon campaign.

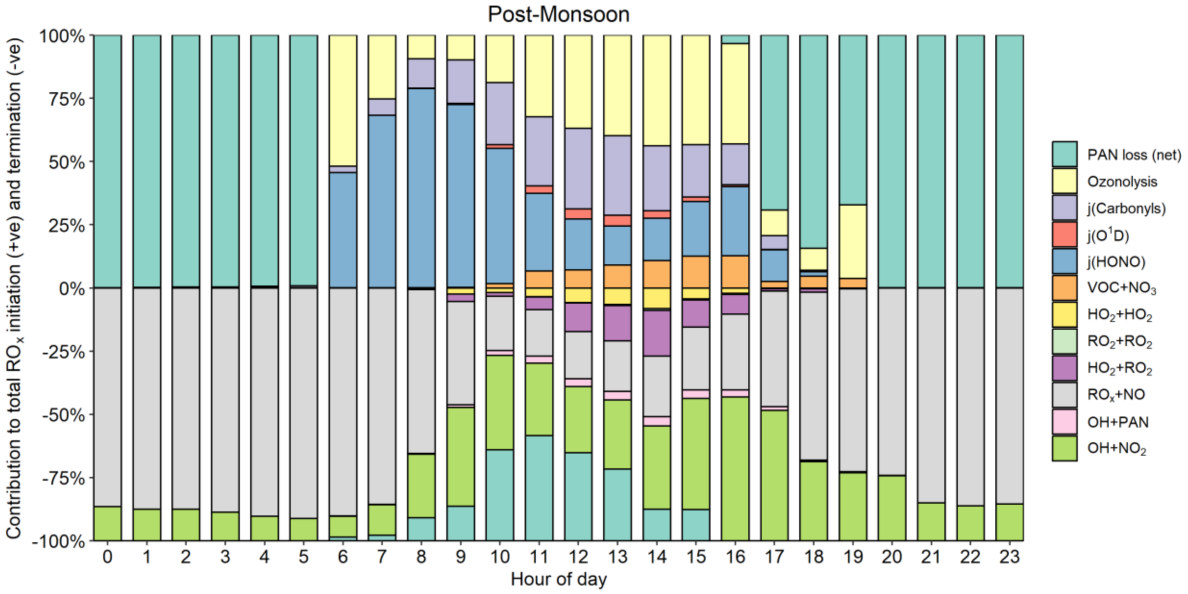

Figure S5: Bar chart showing the hourly averaged modelled contributions to total radical initiation (positive) and termination (negative) during the post-monsoon campaign.

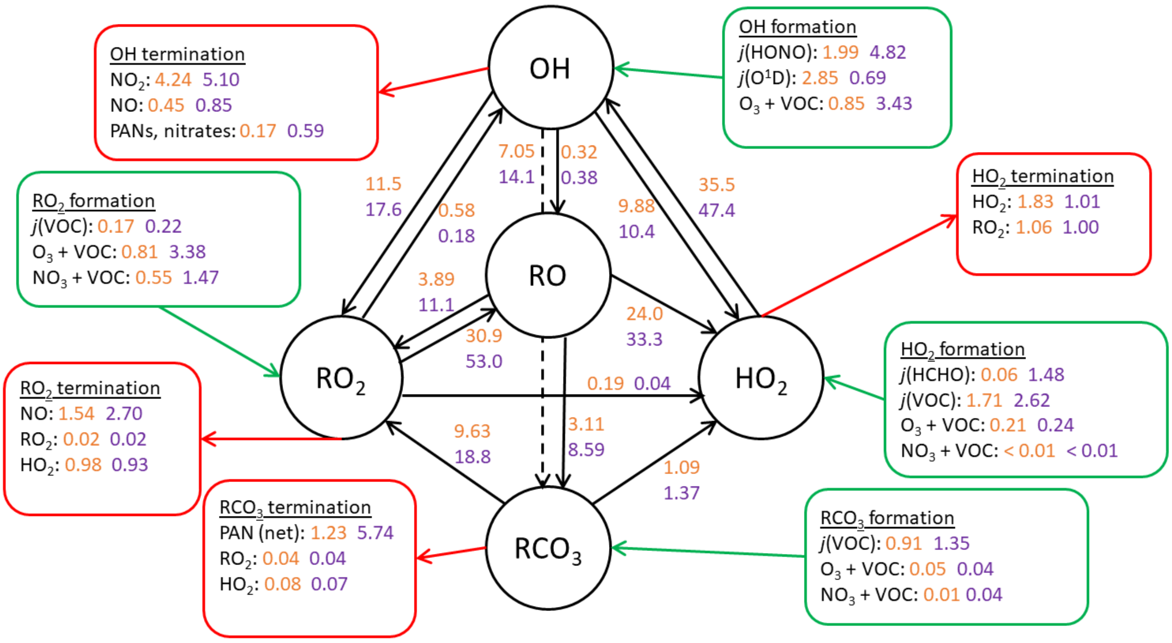

Figure S6: Model reaction flux analysis of the daytime mean rate of reaction for the formation, propagation, and termination of radicals between 11:00 - 15:00 during the pre-monsoon (orange) and post-monsoon (purple) campaigns in ppbV h^{-1}. Radical initiation and termination routes are shown in green and red boxes respectively, where $RCO_3 = RC(O)O_2$.

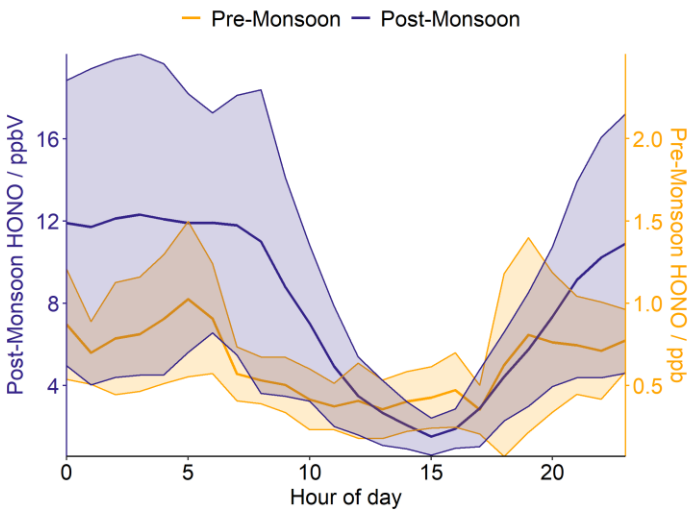

Figure S7: Mean diel HONO mixing ratios during the pre-monsoon (yellow) and post-monsoon (purple) campaigns. The coloured ribbon represents one standard deviation from the mean.

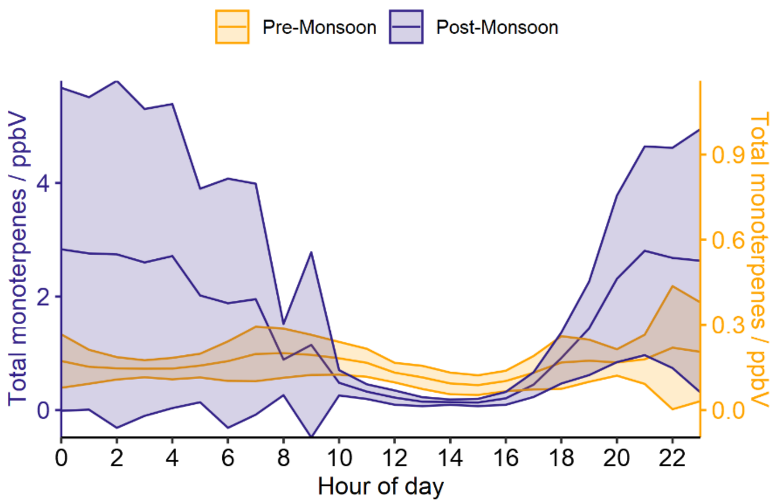

Figure S8: Diel profiles of the mean total monoterpene mixing ratios during the pre-monsoon (orange) and post-monsoon (purple) campaigns. The coloured ribbon represents one standard deviation from the mean.

References:

R. Atkinson, D. L. Baulch, R. A. Cox, J. N. Crowley, R. F. Hampson, R. G. Hynes, M. E. Jenkin, M. J. Rossi, and J. Troe. "IUPAC Task Group on Atmospheric Chemical Kinetic Data Evaluation". In: Atmos. Chem. Phys. 4 (2004), pp. 1461–1738. URL: https://iupac.aeris-data.fr/, last accessed: December 2022.
